# Histological Assessment of PAXgene Tissue Fixation and Stabilization Reagents

**DOI:** 10.1371/journal.pone.0027704

**Published:** 2011-11-16

**Authors:** Marcel Kap, Frank Smedts, Wolter Oosterhuis, Rosa Winther, Nanna Christensen, Bilge Reischauer, Christian Viertler, Daniel Groelz, Karl-Friedrich Becker, Kurt Zatloukal, Rupert Langer, Julia Slotta-Huspenina, Koppany Bodo, Bas de Jong, Uwe Oelmuller, Peter Riegman

**Affiliations:** 1 Department of Pathology, Josephine Nefkens Institute, Erasmus MC, Rotterdam, Netherlands; 2 Dako, Research and Development, Glostrup, Denmark; 3 Department of Pathology, Technical University of Munich, Munich, Germany; 4 Institute of Pathology, Medical University of Graz, Graz, Austria; 5 Qiagen, Research and Development, Hilden, Germany; Institut national de la santé et de la recherche médicale (INSERM), France

## Abstract

Within SPIDIA, an EC FP7 project aimed to improve pre analytic procedures, the PAXgene Tissue System (PAXgene), was designed to improve tissue quality for parallel molecular and morphological analysis. Within the SPIDIA project promising results were found in both genomic and proteomic experiments with PAXgene-fixed and paraffin embedded tissue derived biomolecules. But, for this technology to be accepted for use in both clinical and basic research, it is essential that its adequacy for preserving morphology and antigenicity is validated relative to formalin fixation. It is our aim to assess the suitability of PAXgene tissue fixation for (immuno)histological methods. Normal human tissue specimens (n = 70) were collected and divided into equal parts for fixation either with formalin or PAXgene. Sections of the obtained paraffin-embedded tissue were cut and stained. Morphological aspects of PAXgene-fixed tissue were described and also scored relative to formalin-fixed tissue. Performance of PAXgene-fixed tissue in immunohistochemical and *in situ* hybridization assays was also assessed relative to the corresponding formalin-fixed tissues. Morphology of PAXgene-fixed paraffin embedded tissue was well preserved and deemed adequate for diagnostics in most cases. Some antigens in PAXgene-fixed and paraffin embedded sections were detectable without the need for antigen retrieval, while others were detected using standard, formalin fixation based, immunohistochemistry protocols. Comparable results were obtained with *in situ* hybridization and histochemical stains. Basically all assessed histological techniques were found to be applicable to PAXgene-fixed and paraffin embedded tissue. In general results obtained with PAXgene-fixed tissue are comparable to those of formalin-fixed tissue. Compromises made in morphology can be called minor compared to the advantages in the molecular pathology possibilities.

## Introduction

Formalin has been the fixative of choice for many decades, and as a result, pathology departments have collected vast archives of formalin-fixed and paraffin embedded (FFPE) samples. These archives of well-defined and documented tissue samples are frequently used in medical research [1]. Histomorphology together with immunohistochemistry (IHC) are the foundations on which all diagnostic and pathological research is based. IHC, the most commonly used tool for tumor phenotyping can, with or without application of antigen retrieval, be performed on FFPE sections providing pathologists with both excellent morphology and reproducible results. Multi-center ring trials, however, show that reproducibility of IHC in FFPE tissue is often compromised by the degree of tissue fixation and the various antigen retrieval protocols [2] used in different laboratories (for more information see www.nordiqc.org). The last 20 years molecular diagnostics were added to the histological assays to enable further discrimination of patient groups and subsequent treatment. In our department for molecular diagnosis we noted that fixation level dependent variation limits our ability to apply techniques used in routine diagnostics to molecular research. While (mi)RNA, DNA and proteins can be isolated from FFPE samples [3]–[8], assay reproducibility; hence diagnostic value may be limited. This is due to the poor quality of the derivates caused by varying fixation times which results in high variation of the number of cross-links in any individual sample [9].

Limited possibilities for reproducible application of molecular diagnostics and research on paraffin embedded tissue is one of two major reasons why formalin fixation should be replaced by a non-cross-linking (alcohol based) fixative. The second reason is the alleged carcinogenicity of formalin [10], [11]. Despite the fact that pathology laboratories have invested in advanced air conditioning units to avoid formalin toxicity, formalin substitutes continue to be developed [12]–[14]. An impediment to the wide acceptance of formalin substitutes, however, is the fact that pathologists prefer formalin fixation because they have been trained in the assessment of common artifacts in FFPE tissues. Formalin is also a cheap reagent compared to commercially available alcohol based fixatives. Furthermore, morphology of tissue fixed with non-formalin fixatives is, with few exceptions [15], not the same as the morphology of FFPE tissue.

The European FP7 project SPIDIA (Standardisation and improvement of generic Pre-analytical tools and procedures for In-vitro DIAgnostics), a unique consortium of European universities and biotechnology companies, aims at standardization and improvement of generic pre-analytical tools and procedures for *in vitro* molecular diagnostics. The goal of the consortium is to develop pre-analytical tools for molecular diagnostics which improve the stabilization, handling and study of biomolecules in blood, plasma, serum and tissues. By standardizing pre-analytic and analytic procedures, it is foreseen that the ultimate result of this effort will lead to significant improvement in patient care. We therefore seek to standardize a tissue fixation process that, along with preserving histomorphology, guarantees the extraction of the maximum yield of high-quality nucleic acids from diseased and normal tissue. Along with an improvement in the sensitivity and specificity of routine molecular diagnostic tests, we anticipate that new preanalytical tools for tissue fixation will result in a tissue archive comprised of samples in which high quality biomolecules are preserved. The development of new non-cross-linking fixation reagents, such as the PAXgene tissue fixation and stabilization reagents, therefore, is expected to be pivotal in the standardization of diagnostic and research procedures and may also find applications in biobanking. According to the manufacturer's product description, the PAXgene fixation reagent is a non-carcinogenic, non-cross-linking mixture of different alcohols, acid and a soluble organic compound that rapidly preserves morphology and all bio-molecules. The PAXgene stabilization reagent contains a mixture of alcohols and is applied after fixation. It acts as a storage and transport medium in which morphology and all bio-molecules in the tissue are stabilized before processing. During the development of PAXgene other alcohol based fixatives and transport media like RNA later were tested for both histological and molecular features. PAXgene was the only fixation method which resulted in optimal conditions for both histology and molecular aspects (unpublished observation).

In this paper we compare morphology, histochemistry and antigenicity of PAXgene-fixed and paraffin embedded (PFPE) with formalin-fixed and paraffin embedded (FFPE) tissues. Three independent (no affiliation with any company) pathology labs within the SPIDIA consortium participated in this study, using their own established protocols and automated procedures for tissue processing, H&E staining and IHC procedures. Simultaneously, two other pathology labs within the SPIDIA consortium worked on the proteomic and genomic assessments of the PAXgene fixative. The proteomic data are published elsewhere [16] and the manuscript reporting stabilization of nucleic acids in PAXgene-fixed and stabilized tissue is submitted for publication.

## Materials and Methods

### Human tissue collection

Tissue samples used for this study were unfixed specimens, collected during routine grossing at the respective pathology departments of the Erasmus MC (Rotterdam, The Netherlands)(EMC), the Medical University Graz (Graz, Austria)(MUG) and the Technical University Munich (Munich, Germany)(TUM). Tissue was collected with consent according to each institute's local legislation and policies. EMC declares the following; The use of residual tissue accompanied by data on tissue type and disease state is approved by the Erasmus MC Medical Ethical Commission under number MEC-2008-397. Since the Dutch Code of Conduct legislation concerning the use of residual tissue for research is adhered to, no informed consent was necessary for this work. TUM declares the following; All patients gave written informed consent, and the study was approved by the Ethics Committee of the Klinikum rechts der Isar of the Technische Universität München, Germany (reference number 2336/09). MUG declares the following; All sample donors provided written informed consent and the study was approved by the Ethics Committee of the Medical University of Graz, Austria (reference number 20-066).

For each tissue studied, a 4 mm thick tissue specimen was removed from an organ or tumor. This specimen was then subdivided into three approximately equal samples for treatment by either formalin or PAXgene or to be snap-frozen in liquid nitrogen (LN2). Frozen tissue samples were archived for reference in proteomic and genomic research. The following tissues were collected: adrenal gland (1), bladder (2), bonemarrow (5), colon (4), colon cancer (1), esophagus (2), fat (5), fibroma (1), kidney (3), renal cancer (1), liver (9), lung (4), lymphoma (3), breast cancer (2), striated muscle (2), myoma (2), ovarian carcinoma (3), pancreas (1), placenta (1), prostate (2), rectum (5), skin (4), small intestine (4), spleen (2), soft tissue (1), stomach (6), testis (1), thyroid (2) and uterus (1).These were fixed either in formalin for 24 hours (4% neutral buffered formaldehyde) or in PAXgene tissue fixation reagent (Paxgene Tissue System, PreAnalytix GmbH, Hombrechtikon, CH) for either 3 or 24 hours. After fixation in PAXgene tissue fixation reagent, tissues were transferred to PAXgene tissue stabilizer reagent and stored for a minimum of 24 hours or up to one week at room temperature. PAXgene treated samples were processed in a formalin-free tissue processor (ASP3000, Leica). Six changes, 1 hour each, of 100% ethanol and 3 changes of xylene, 1 hour each, were applied in a vacuum at room temperature before tissue was impregnated with 3 changes of low melting point paraffin at 60°C for no longer than 3 hours. Bonemarrow samples were decalcified by submerging the tissue in 10% formic acid (Merck, Germany) for 48–72 hours. Other participating laboratories used essentially the same processing protocol but with different processing instruments.

Tissue samples from human breast cancer were acquired with informed consent from Cureline Inc. (San Francisco, USA). After resection, tumor specimens were divided, one part fixed in neutral buffered formalin (NBF) for 24 h at room temperature, and the other part fixed and stabilized in the PAXgene Tissue Container (Cat.# 765112, PreAnalytiX, Hombrechtikon, Switzerland). Fixation was performed at room temperature in chamber 1 of the container. Fixation was stopped after 2–4 hours by transfer into chamber 2. Samples were stored for up to 5 days at 4°C until they were processed manually with 80%, 90%, 95%, 99% ethanol(2×), followed by isopropanol (2×), xylene (2×), and infiltration and embedding in low-temperature melting paraffin. Formalin-fixed samples were processed according to Cureline SOP with 70%, 80%, 90%, 100% (3×) alcohol, xylene (3×), and infiltration and embedding with high-grade melting paraffin. All blocks of formalin-fixed, paraffin-embedded (FFPE) and PAXgene-fixed, paraffin-embedded (PFPE) tissues were stored at 4°C in the dark until use.

### H&E staining

Sections of FFPE and PFPE tissue, 4 µm thick were cut on a microtome (HM335E, Microm GmbH, Germany). Slides were dried for 15 minutes at 42°C on a slide warmer (Slide warmer SW85, Adamas Instruments B.V., The Netherlands). In our department H&E staining was performed according to a routine standard operating procedure using a Leica Multistainer (ST5020, Leica). Slides were dewaxed and rehydrated with successive applications of xylene, alcohol 100%, alcohol 70% and tap water. Haematoxylin (Mayer's, Klinipath, Benelux) was applied for 4 minutes followed by a 20 second differentiation in ammonia after which eosin (Eosin Y A+B, Klinipath, Benelux) was applied for 20 seconds. At the other laboratories H&E staining was performed using similar protocols, but with different reagents and machines. Only one H&E stained slide was prepared from each FFPE or PFPE tissue sample.

### Virtual microscopy

To ensure that all pathologists examined the exact same tissue section, slides were completely digitized using the Hamamatsu Nanozoomer Digital Pathology (NDP) slide scanner (Hamamatsu, Japan) at a resolution of 40× which is comparable to 400× magnification on a microscope. The areas to be scanned and focus points were manually set. The obtained files were uploaded into a secured internet environment to which the pathologists had access. NDP Slideviewer software; NDP server and NDP View, provided by Hamamatsu, were used to score morphologic features. Images shown in this paper are sections of the files exported from the digital slides.

### Morphology scoring

This part of the study was not performed blind to the fixative, because the primary goal was to evaluate potential morphological artifacts of PAXgene-fixed tissues. Four pathologists were asked to assess and compare overall morphology, contrast and nuclear, cytoplasmic, membrane detail and the most obvious fixation artifacts on paired PFPE and FFPE slides. Morphology of tissue fixed for 24 hours in formalin was defined as the baseline (score = 0). Five grades were used to compare PFPE morphology to FFPE morphology. PFPE morphology could be equal to (0), better (1), considerably better (2), worse (−1) or considerably worse (−2) than FFPE morphology. “Considerably worse” was considered unacceptable for diagnostic purposes, “Worse” is considered to represent poor quality, but nonetheless acceptable for diagnostic purposes. Scores from all pathologists were recorded for tissue type, fixation method and duration. The average score and standard deviation for each tissue type were calculated (see table 1) in order to depict the relative performance of PAXgene tissue fixation reagent and inter-observer variance in one graph.

**Table 1:** 

	Pathologist TUM1		Pathologist TUM2		Pathologist MUG		Pathologist EMC	
	*average score*	*st. dev*	*average score*	*st. dev.*	*average score*	*st. dev.*	*average score*	*st. dev.*
all scores	0.16	0.44	0.16	0.56	−0.05	0.60	0.13	0.61
scores EMC slides	0.14	0.47	0.10	0.50	0.07	0.66	0.04	0.66
scores MUG slides	0.22	0.42	0.21	0.67	−0.15	0.40	0.17	0.52
scores TUM slides	0.17	0.38	0.31	0.54	−0.28	0.59	0.41	0.50

### Histochemical stains

Frequently used histochemical and histological staining procedures were selected for assessment of PFPE tissue. Periodic acid schiff (PAS) and resorcin fuchsin (RF; elastin stain) are commonly used histochemical stains, sirius red (SR; collagen stain) is a typical histological stain, and Gomori (GOM; reticulin stain) represents a silver-gold enhanced histochemical staining procedure.

RF and SR stains were performed according to standard FFPE based protocols [17]. PAS and GOM stains were performed using an Artisan stainer (Artisan, DAKO, Denmark) and DAKO Artisan staining reagents (Artisan PAS kit, AR165, DAKO, Denmark and Artisan reticulin-Nuclear Fast Red kit, A179, DAKO, Denmark). Sections, 4 µm thick, of FFPE and PFPE colon tissue were dewaxed and rehydrated (except sections for RF staining, which were dewaxed and stored in 70% ethanol) before staining.

### IHC

Sections, 4 µm thick were cut from PFPE and FFPE tissue blocks right after embedding and after two years of room temperature storage (IHC with CD3, CD20, CD68, KERPAN, KER8.18 and S100 was repeated on blocks that were stored for two years). PFPE and FFPE sections were mounted on the same glass slide to ensure equal treatment of all sections. The slides were dried for 15 minutes at 42°C and baked at 65°C for 15 minutes to enhance adherence. For the first PFPE IHC assessment, we subjected the slides to standard (as used for FFPE) heat induced epitope retrieval (HIER). To demonstrate any changes in the level of antigen masking due to absence of cross-links, we also omitted HIER. At Erasmus MC, HIER was performed using a DAKO PT module (PT Link, Dakocytomation, Denmark) with DAKO PT High pH Buffer (Envision FLEX target retrieval solution high pH 50×, K8004, Dakocytomation, Denmark) at 99 degrees centigrade for 15 minutes. For DAKO PharmaDX EGFr IHC the slides were treated with proteinase K (5 minutes at room temperature) provided with the ready to use kit (EGFR PharmDX For Autostainer, K1494, Dakocytomation, Denmark). The IHC procedure was performed using a DAKO Stainer (DAKO Autostainer plus, Dakocytomation, Denmark). At Erasmus MC the sections were incubated with PBS/3%H_2_O_2_ for ten minutes to block any endogenous peroxidase activity. Slides were incubated with primary antibodies for 30 minutes followed by DAKO envision conjugate for 30 minutes. The conjugate's peroxidase label was visualized by DAB+ (DAKO Real, K5007, Dakocytomation, Denmark). Slides were counterstained with Mayer's Heamatoxylin (Mayer's, Klinipath, Benelux) for 1 minute, dehydrated and a cover slip applied (Leica multistainer/coverslipper station, resp. ST5020 and CV5030, Leica). The different antigen retrieval methods of partner labs and IHC protocols are listed in table 2. Specificity of IHC stains from both untreated and HIER treated PFPE sections were compared to the FFPE sections. If immunohistochemical reactions in PFPE sections were different to those in FFPE sections, only the antibody dilution was altered except for the progesterone receptor antibody for which another clone was used to replace the non-reacting clone.

**Table 2:** 

Antibody	Clone	Company and code number	Dilution	Antigen retrieval	
				*PAXgene fixation 3 hours*	*Formalin fixation 24 hours*
BCL-2	124	DAKO M0887	1∶100	DAKO PT Hi 15 minutes	DAKO PT Hi 15 minutes
BCL-6	GI191E/A8	ITK CMC796	1∶100	DAKO PT Hi 15 minutes	DAKO PT Hi 15 minutes
CD2	AB75	MONOSAN MONX10830	1∶50	DAKO PT Hi 15 minutes	DAKO PT Hi 15 minutes
CD3	polyclonal	DAKO A0452	1∶150	none	DAKO PT Hi 15 minutes
CD4	4B12	MENARINI 35066	1∶10	DAKO PT Hi 15 minutes	DAKO PT Hi 15 minutes
CD5	4C7	MONOSAN MONX10335	1∶100	DAKO PT Hi 15 minutes	DAKO PT Hi 15 minutes
CD7	CBC.37	DAKO M7255	1∶10	DAKO PT Hi 15 minutes	DAKO PT Hi 15 minutes
CD8	C8/144B	DAKO M7103	1∶200	DAKO PT Hi 15 minutes	DAKO PT Hi 15 minutes
CD10	56C6	MONOSAN MONX10354	1∶20	DAKO PT Hi 15 minutes	DAKO PT Hi 15 minutes
CD20	L26	DAKO M0755	1∶400	none	DAKO PT Hi 15 minutes
CD21	1F8	DAKO M0784	1∶30	DAKO PT Lo 15 minutes	DAKO PT Lo 15 minutes
CD23	SP23	Neomarkers RM-9123-S	1∶25	DAKO PT Lo 15 minutes	DAKO PT Lo 15 minutes
CD31	JC70A	DAKO M0823	1∶50	DAKO PT Hi 15 minutes	DAKO PT Hi 15 minutes
CD68	KP-1	DAKO M0814	1∶1600	none	DAKO PT Hi 15 minutes
CD79a	JCB117	DAKO M7050	1∶100	DAKO PT Hi 15 minutes	DAKO PT Hi 15 minutes
CD117	YR145	Cellmarque 117R-16	1∶50	DAKO PT Hi 15 minutes	DAKO PT Hi 15 minutes
D2-40	D2-40	DAKO M3619	1∶50	DAKO PT Hi 15 minutes	DAKO PT Hi 15 minutes
EGFR	2-18C9	DAKO K1494	R.T.U.*	protK 5 minutes R.T.	protK 5 minutes R.T.
ER	1D5	DAKO M7047	1∶35	TE pH9 98°C 20 minutes	TE pH9 98°C 20 minutes
glycophorin C	RET40f	DAKO M0820	1∶600	DAKO PT Hi 15 minutes	DAKO PT Hi 15 minutes
Her2Neu	Herceptest	DAKO K5204	R.T.U.*	Kit buffer 98°C 40 minutes	Kit buffer 98°C 40 minutes
Her2Neu	4B5	Roche, 790-2991	R.T.U.*	HIER cell conditioner (Benchmark)	HIER cell conditioner (Benchmark)
Inhibin	R1	DAKO M3609	1∶50	DAKO PT Hi 15 minutes	DAKO PT Hi 15 minutes
KER5/6	D5/16B4	DAKO M7237	1∶50	HIER cell conditioner (Benchmark)	HIER cell conditioner (Benchmark)
KER8&18	5D3	Neomarkers MS 743-S	1∶100	none	DAKO PT Hi 15 minutes
KER-PAN	AE1/AE3	Neomarkers MS 343-P	1∶200	DAKO PT Hi 15 minutes	DAKO PT Hi 15 minutes
KI-67	MIB-1	DAKO M7240	1∶100	DAKO PT Hi 15 minutes	DAKO PT Hi 15 minutes
OCT3/4	C-10	Santa Cruz sc-5279	1∶80	DAKO PT Hi 15 minutes	DAKO PT Hi 15 minutes
P53	DO-7	DAKO M7001	1∶400	DAKO PT Hi 15 minutes	DAKO PT Hi 15 minutes
PLAP	8A9	DAKO M7191	1∶100	DAKO PT Hi 15 minutes	DAKO PT Hi 15 minutes
PR	1A6	Bioprime PR500	R.T.U.*	TE pH9 98°C 20 minutes	TE pH9 98°C 20 minutes
S100	polyclonal	DAKO Z0311	1∶3200	none	DAKO PT Hi 15 minutes
Vimentin	V9	DAKO M0725	1∶1000	DAKO PT Hi 15 minutes	DAKO PT Hi 15 minutes

*R.T.U. = ready to use.

### HER2 IHC

Sections of PFPE and FFPE human breast cancer tissue with a thickness of 4 µm were mounted on SuperFrost Plus slides (Cat.# 631-0108, VWR, Darmstadt, Germany). Immunohistochemistry for determination of HER2 protein overexpression was performed with the HercepTest kit (Code K5204, Dako, Glostrup, Denmark). All samples were treated according to manufacturer's instructions with the exception of the epitope retrieval step with PFPE sections. In the case of FFPE tissue, the sections were placed in a staining dish filled with epitope retrieval solution (vial no. 7, HercepTest kit) and incubated for 40 minutes at 98°C in a water bath. PFPE tissue sections were incubated for 5 minutes at 98°C in a pH9 target retrieval solution (Code S2367, Dako, Glostrup, Denmark). Counterstaining was done with all samples for 2 minutes with hematoxylin.

### HER2 CISH

For determination of HER2 gene amplification sections of FFPE and PFPE human breast cancer samples with a thickness of 6 µm mounted on SuperFrost Plus slides (Cat.# 631-0108, VWR, Darmstadt, Germany) were de-waxed, pretreated, and hybridized with reagents from the SPOT-Light HER2 CISH kit (Cat.#84-0150, Invitrogen Corporation, Camarillo, USA). Sections from FFPE tissue were processed according to manufacturer's instructions including heat treatment and a 5 minutes protease digestion step. For PFPE tissue sections, the heat pretreatment and enzyme digestion steps were omitted. After deparaffinization, sections were air-dried and covered with the HER2 probe for denaturation and hybridization. Counterstaining was performed with all samples for 5 seconds with hematoxylin (Reagent J, SPOT-Light HER2 CISH kit).

## Results

### Morphology

**Figure 1:**
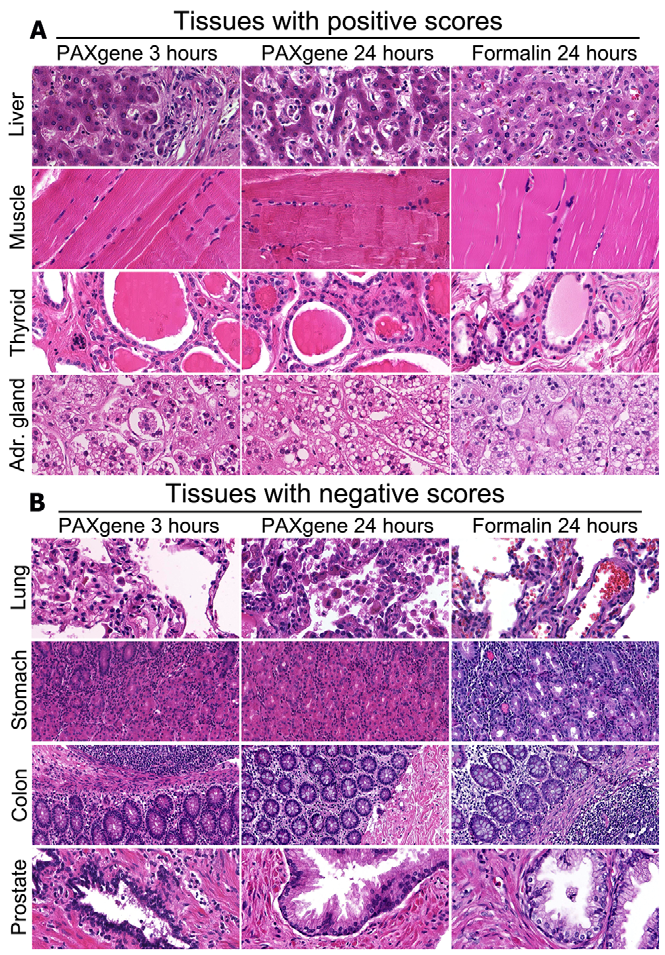
Morphology of various normal tissues. Figure 1a shows H&E stains in tissues fixed for 3 hours in PAXgene (left column), 24 hours in PAXgene (centre column) and for comparative purposes routine fixation in formalin for 24 hours (right column). In liver PAXgene fixation scores better on contrast. Striated muscle shows more detail after PAXgene fixation. Thyroid tissue scores better in all scored details. And adrenal gland tissue predominantly scores better in membrane detail. (400× original magnification). Figure 1b shows H&E stains in tissues fixed for 3 hours in PAXgene (left column), 24 hours in PAXgene (center column) and for comparative purposes routine fixation in formalin for 24 hours (right column). Lung tissue appears swollen. In gastric tissue the cell differentiation is harder to distinguish. In colon the distinctive granules in Paneth cells are less easily detectable after PAXgene fixation and in prostate the contrast of the epithelium is lower in PFPE tissue compared to FFPE tissue. (Lung and prostate 400×; gastric and colon tissue 200× original magnification).

In the evaluation of morphology of human tissue, the morphology of PFPE tissue appeared clearly comparable to FFPE tissue (see figure 1a). Morphology of PFPE liver was crisper and showed slightly more contrast as compared to FFPE tissue. In striated muscle tissue, contrast is also slightly enhanced after PAXgene fixation. The same evaluation was noted for thyroid and adrenal gland tissue in which the contrast between FFPE and PFPE is somewhat stronger in the PAXgene-fixed tissues. Overall, the differences between FFPE and PFPE tissues were deemed to be minimal. Elevated levels of eosinophilia, while noted in PFPE tissues, did not seem to hinder observations. PFPE lung tissue appears swollen in relation to FFPE lung tissue. This may be due to the fact that red blood cells (RBC), normally found in and around alveoli are damaged and appear empty, or are, in some cases, completely lysed in PFPE tissue. In the lung, only minor variations are observed with slightly less contrast in the lung tissues after PAXgene fixation compared to formalin fixation. In tissues from stomach, prostate and small intestine PAXgene tissue fixation reagent seems to cause small deviations in morphology (see figure 1b). In stomach tissue, the PAXgene fixation shows less contrast between glands and infiltrate, and discrimination between parietal and chief cells is difficult. In the colon, morphology of PFPE and FFPE tissues is nearly identical, but Paneth cells are less readily identified after PAXgene fixation. In prostate, PAXgene fixative causes slight pyknosis of nuclei and minor cell shrinkage compared to formalin fixation.

**Figure 2:**
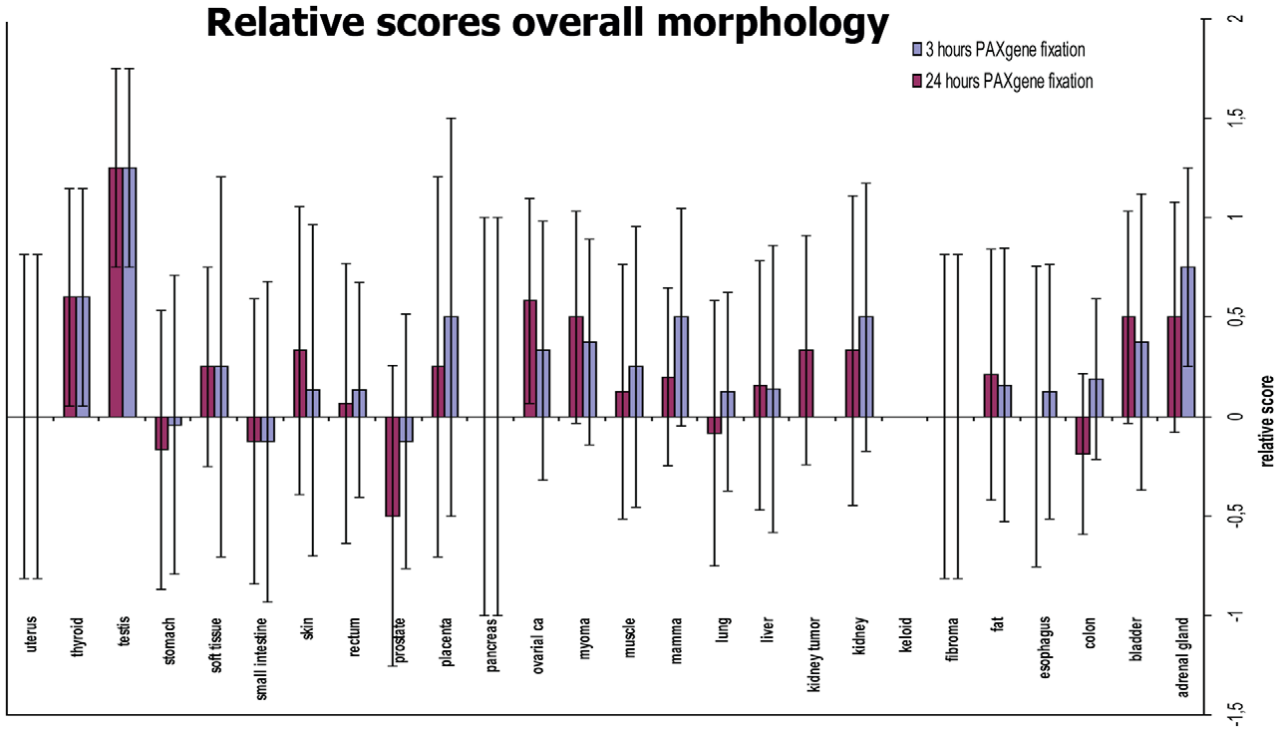
Relative morphology scores of human tissues. In this graph relative scores of overall morphology of 3 hours fixation and 24 hours fixation are compared. All PAXgene-fixed tissue is scored relatively to 24 hours formalin-fixed tissue (zero base line). Error bars, representing inter-observer variance, are shown.

The overall scores of the H&E staining evaluation by four pathologists are presented in a comprehensive overview in figure 2. For gastric tissues, the detailed scores are all better for PAXgene fixation and only the overall morphology scores are negative. In small intestine, there is a decrease in the cytoplasmic and membrane details in PFPE tissue that results in this overall negative score, whereas for prostate the only feature better for PAXgene fixation is contrast. Figure 2 shows that, considering the standard deviation, the differences between 3 hours and 24 hours fixation in PAXgene are minor. In general, the variation in estimating various tissue features indicates that observation of morphology is quite subjective. No serious statistical analysis was possible with this number of data points. The average and standard deviation were calculated to indicate the subjectivity of these observations. Except for pathologist TUM2, pathologists do not prefer their trusted in-house H&E stain above H&E stains from other sites. One pathologist (MUG) seems to be very critical, giving mostly negative scores for PFPE tissue, however the overall morphology scores, both positive and negative, are mostly close to the neutral baseline, indicating only minor differences between PFPE and FFPE (table 1).

**Figure 3:**
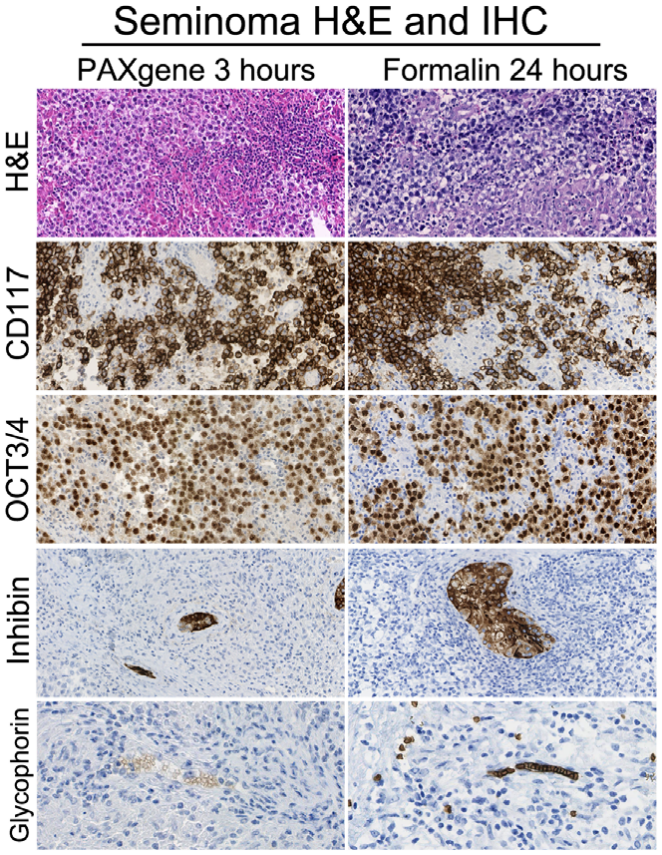
Despite of morphological differences, diagnosis can be confirmed by immunohistochemistry. The left column shows PAXgene-fixed sections, the column on the right shows formalin-fixed sections. Although PAXgene-fixed seminoma tissue seems more compact and cells tend to be rounder and less interconnected the membranes, stained with CD117, are intact. The nuclear antigens OCT3/4 are well preserved. IHC was not optimized for this antibody, therefore the staining is somewhat lighter. The Inhibin stain shows that this particular cytolplasmic antigen is well preserved and did not leak out of the cells. The red blood cells in PAXgene-fixed tissue are damaged, but still express some Glycophorin C. (200× original magnification).

Since red blood cells (RBC) are damaged during PAXgene fixation, we examined seminoma tissue (seminoma tumor cells are fragile cells which barely express any intermediate filaments [18]), in order to investigate possible limitations of the PAXgene tissue fixative. PAXgene fixation did not damage or lyse the seminoma cells. PFPE seminoma tissue did, however, show some dissociation and slightly decreased membrane detail. Immunohistochemistry assessment, however, showed that membranes, cytoplasmic as well as nuclear, in PFPE seminoma tissue express all the antigens also found in regular, FFPE based IHC. The staining patterns show that the cell membranes are intact and that the dissociation did not lead to structural or molecular integrity loss. Furthermore, Glycophorin C was stained to show that, although RBC appear to be empty, the membrane antigen Glycophorin C is still present on RBC (figure 3).

**Figure 4:**
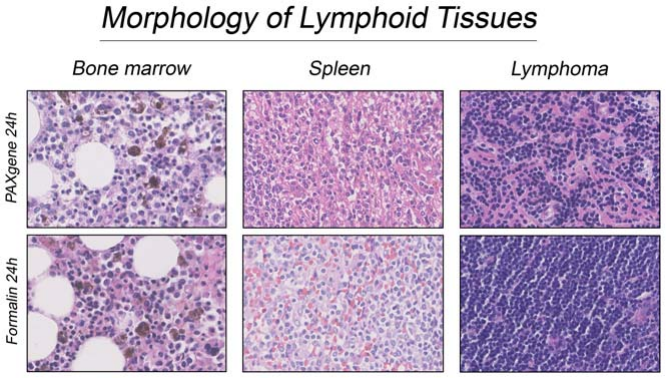
Morphology of lymphoid tissues. The left panel shows bonemarrow after PAXgene or formalin fixation, followed by decalcification in 10% formic acid. The middle panel shows that the PAXgene specific artefacts like red blood cell lysis, aberrant nuclear details and higher eosiniphilia result in a very different picture compared to FFPE tissue. The PFPE lymphoma tissue in the right panel is slightly over stained with eosin which negatively influences the differentiation of hematopoetic lineages. (H&E, 400× original magnification).

The above described artefacts raised the question whether hematopathological diagnostic would be hindered by PAXgene fixation of lymphoid tissue. Red blood cells are often used as internal cell size reference, differentiation of the lymphoid and myeloid lineages needs proper nuclear details and normal hematoxylin/eosin contrast. In spleen tissue the adverse effects of PAXgene fixation became apparent. We observed loss of red blood cells, alteration of nuclear detail and disproportionate cell shrinkage. These artefacts resulted in such morphological changes that the pathologist needed to re-evaluate the cell types by comparing the PAXgene-fixed tissue directly with the formalin-fixed section. Morphology of PFPE bonemarrow and lymphoma samples was comparable to that of FFPE tissue. However, the higher eosinophilia made recognition of the different lineages difficult (figure 4).

### Immunohistochemistry

**Figure 5:**
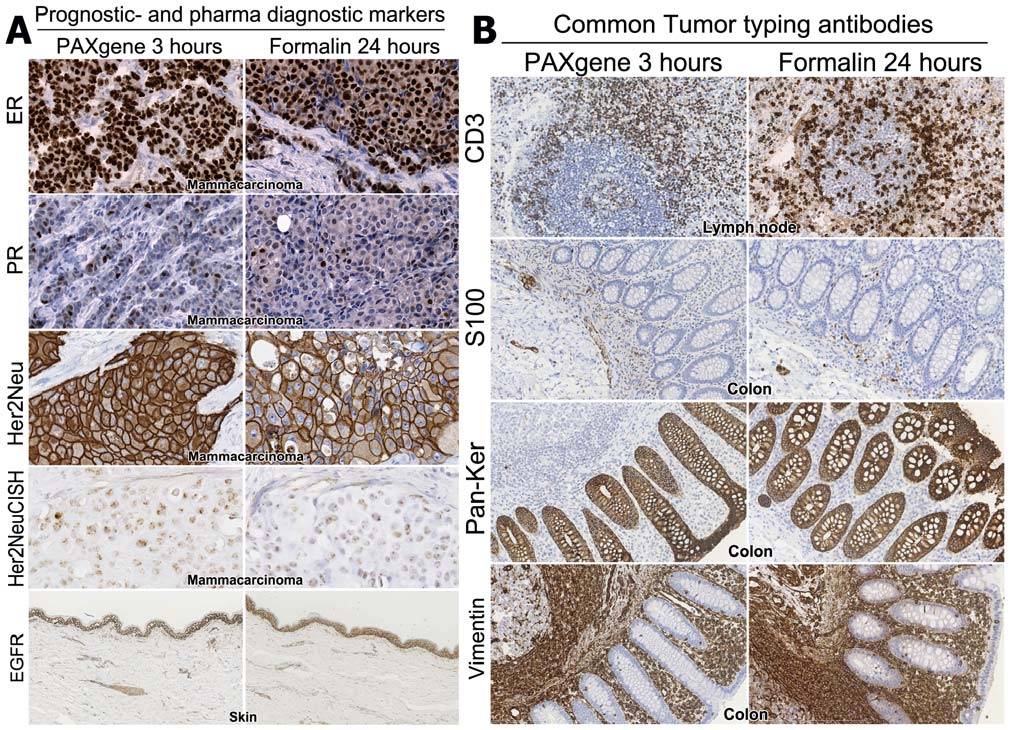
Immunohistochemistry. In figure 5a the most common prognostic (ER and PR) and pharmaco diagnostic markers (Her2Neu and EGFR) are shown. ER, PR are stained in serial sections of the same breast tumor sample. Her2Neu IHC as well as CISH was performed on a breast cancer sample with Her2Neu over expression and gene amplification. EGFR is stained in skin samples. The left column shows PAXgene-fixed sections, the column on the right shows formalin-fixed sections. (ER, PR, Her2Neu 400×; EGFR 100× original magnification). In figure 5b the most common tumor differentiation markers are shown. CD3 is stained in lymph node. S100, Pan-Keratin and vimentine are stained in colon tissue. The left column shows PAXgene-fixed sections, the column on the right show formalin-fixed sections. (200× original magnification).

Of all routinely used immunohistochemical antibodies tested, only a few exhibited lower levels of immune reactivity on PFPE sections compared to mirrored FFPE sections. This was overcome by omitting antigen retrieval, adjustment of antibody concentration, or using another clone (see table 2 for details). Antigen retrieval could be omitted for the following antibodies: CD3, CD20, CD68, KER8.18 and S100. For S100 there was no clear difference in immuno staining with or without antigen retrieval. Neuroendocrine cells in colon mucosa were intensely and specifically stained regardless of whether or not antigen retrieval was applied. The IHC procedure on PFPE tissue with antibodies: BCL-2, BCL-6, CD2, CD4, CD5, CD7, CD8, CD10, CD21, CD23, CD31, CD117, D2-40, ER, EGFR, Her2Neu, PR, glycophorin-C, Inhibin, Ker5/6, KERPAN, KI-67, OCT3/4, P53, PLAP and vimentin still required an antigen retrieval procedure. Typical examples of IHC stains used for determination of prognosis or pharmacodiagnostics are shown in figure 5a. Both nuclear antigens ER and PR were stained equally well in breast carcinoma after both modes of fixation. PR clone PgR636 (DAKO, M3569), however, needed to be replaced by clone 1A6 (Bioprime, PR500) to obtain these results. HER2NEU was stained in 5 different breast tumor samples. The HER2NEU staining pattern in PFPE sections did not always resemble the pattern found in mirrored FFPE sections. In general the PFPE HER2NEU signal is stronger and crisper compared to the signal found in FFPE sections. EGFR in skin looks somewhat more granular in PFPE sections compared to FFPE sections, nevertheless the signal is specifically located in the epidermis and in hair follicles. Commonly used antigens for tumor typing are shown in figure 5b. CD3 is stained in a lymph node and although the staining intensity is somewhat lower in PFPE tissue than in FFPE tissue, the signal is specifically found in all T-cells. S100 stains neuroendocrine cells in colon mucosa. Epithelial cells in colon tissue are stained positive for pankeratin. In colon, almost all cell types are vimentin positive. Epithelium however, is vimentin negative. To investigate epitope stability in PFPE blocks, IHC of CD3, CD20, CD68, KERPAN, KER8.18 and S100 was repeated on blocks that were stored for two years. No deterioration of antigenicity was observed.

### Histochemical stains

**Figure 6:**
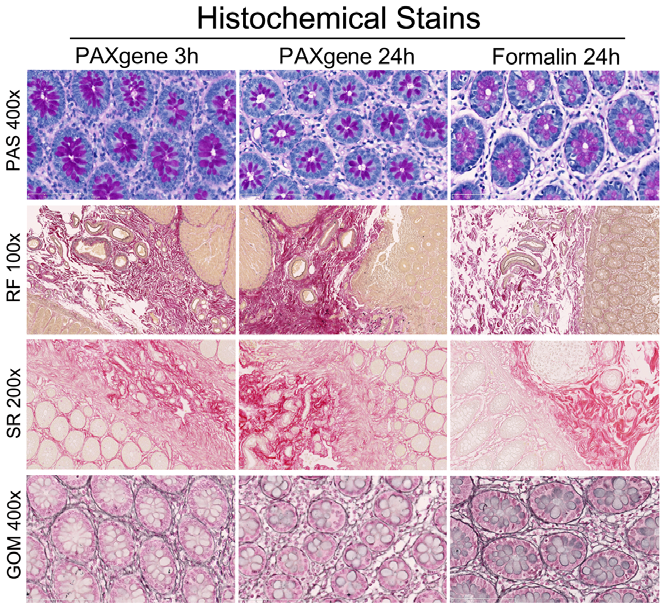
Histochemical stains show some qualitative differences between PAXgene and formalin fixation. In PAXgene-fixed tissue PAS staining results in more condense staining of Goblet cells in colon. The RF, elastin stain of PFPE tissue is similar to the staining pattern in FFPE tissue. The SR, collagen stain in PFPE tissue is stronger compared to the stain in FFPE tissue. The GOM, reticulin stain is crisper in FFPE tissue. In PFPE tissue short fixation results in a stronger, crisper stain compared to longer PAXgene fixation. (PAS and GOM 400×; RF 100× and SR 200× original magnification).

The results of histochemical stains are depicted in figure 6. Different levels of staining intensity were observed between the two fixation methods. After PAS staining, the goblet cells in colon are more intensely stained in PFPE sections as compared to FFPE sections. The staining intensity of basal membranes in blood vessels, however, is the same in both fixation methods. This indicates that specifically mucin in Goblet cells is more condensely present after PAXgene fixation. The RF stain, which in FFPE results in black elastin fibers, red collagen fibers and yellow muscle fibers, shows the same results in PFPE tissue. However, after 3 hours of PAXgene fixation Goblet cells are stained light purple. This stain is less distinct when tissue is fixed in PAXgene for 24 hours. Collagen fibers were stained with the SR staining method. Collagen fibers are stained red, whereas muscle fibers are stained yellow. In PFPE sections the collagen fibers between the crypts appear crisper than in FFPE tissue. In FFPE sections, this pattern is also present, but the intensity is much lower. The reticulin stain (Gomori silver impregnation) shows that reticulin fibers are stained less intensely in PFPE sections as compared to FFPE sections. The staining in PAXgene tissue fixed for 3 hours is crisper than that of tissue fixed for 24 hours PAXgene.

### HER2 CISH

Her2Neu CISH is possible on PFPE breast cancer tissue. Figure 5a shows that the breast cancer tissue (ductal *in situ* carcinoma) which also over-expresses the Her2Neu protein shows Her2 gene amplification. Although the signal in the PFPE section is stronger as compared to the signal in the FFPE section, the results show that in both sections HER2NEU gene amplification is present.

## Discussion

The results of our exploratory investigation clearly show that despite slight differences seen between FFPE and PFPE tissues, PAXgene tissue fixation and stabilization reagents indeed preserve morphology, histochemical features, and antigenicity such that the reagents can eventually be used in routine pathology laboratories. Molecular diagnostics and medical research can benefit from this fixative, because (described in a separate publication) it appears to preserve the macromolecules in the tissue in a more native state than in tissue fixed with formalin [16]. All diagnostic techniques described in this paper can be performed on PFPE material without major protocol adaptations. Furthermore, a future PFPE archive may provide researchers with high quality and easily accessible bio-molecules which can be harvested from the same morphologically well described and diagnosed tissue blocks [16]. For contemporary basic pathology research, this means that it would no longer be necessary to divide small specimens into two even smaller samples: one for morphology and IHC (FFPE) and another for molecular research (snap freezing or immediate RNA isolation). PAXgene seems to provide the answers to many questions, but in the meantime, some critical questions are raised.

According to participating pathologists, the most salient feature of PFPE tissue was the quality of the H&E staining. They noted that in general, the staining for both haematoxylin and eosin were more intense in the PFPE tissues than in corresponding FFPE tissues. In many cases this effect resulted in increased contrast between the components of the various tissues. Details such as nuclear and cytoplasmic detail were also affected by the choice of fixative. Surprisingly however, these details were variously scored as either positive or negative factors in the evaluation of tissue morphology. This indicates that the individual pathologist can sense that there is a shift in morphological features, but that it is difficult to evaluate whether this has a definitive positive or negative effect on how they evaluate tissue. It is known that morphologic evaluation of tissue is subject to considerable inter- and intra-observer variability, therefore, it could be that the effect of the PAXgene fixation on tissue morphology, despite the fact that it is unequivocally observed, falls within the range of inter- and intra-observer variability in general. Of course this is all speculative, since we chose not to perform a blind, but a comparative study. This approach uncovered a number of morphologic features characteristic of PAXgene fixation that are not seen in FFPE tissue, the most noticeable of which are the loss of granules in Paneth cells and fragmentation of red blood cells. This effect is probably due to the acetic acid component of the fixative which seems to dissolve some constituents of cytoplasm or cell membrane. Whether these artifacts influence the establishment of a proper diagnosis in cancer tissue will be further investigated during a world wide SPIDIA ring trial, planned in the near future. One problem mentioned by the hematopathologist is that red blood cells are often used as a cell size reference in lymphoma diagnostics. Since the cell shrinkage factor in PFPE tissue seems slightly different opposed to FFPE tissue, a new reference is needed. Endothelial cells could be of use, since the capillaries in PFPE tissue are more open and easily detected. The nuclear detail in lymphoid cells in spleen tissue is slightly different in PFPE tissue compared to FFPE tissue. This effect underlines the need for a new learning curve for pathologists to get acquainted with the PAXgene fixation induced artefacts.

PAS, reticulin, elastin and sirius red stains were performed to investigate whether the appearance of histochemical stains on PFPE tissue and FFPE tissue were similar. The protocols used for these stainings were not optimized for use with PAXgene-fixed tissue. However, follow up experiments showed certain tissue substances stained during these procedures react differently to PAXgene fixation compared to formalin fixation. The findings do, however, indicate that with some fine-tuning of protocols the PFPE results can become the same as the standard FFPE result. Considering that different pathology labs use different protocols they will need to adopt their own workflow.

The results also show that immunohistochemistry in PFPE sections is very comparable to IHC in FFPE sections. We observed some limitations which needed further investigation. The progesterone receptor antibody had to be replaced by an antibody from a different clone to obtain results seen in FFPE sections. Antigens remain stable in the tissue blocks for at least two years. When IHC was repeated on two year old blocks, the specificity and sensitivity where comparable to those of fresh blocks. Chromagen in situ hybridization (CISH) was performed without any problems, whereas fluorescence in situ hybridization (FISH) needs further investigation. Because DNA is more natively preserved it is easier accessible for the used probes, even without protease digestion or cooking the slides. Without protein digestion (PFPE tissue is more prone to over-digestion) auto fluorescence is a major obstacle in some tissue types. Cooking the slides did improve the signal to noise ratio, but results are not yet optimal. Specific tumor types are now being collected to study the effectiveness of improved FISH protocols.

Like Kryofix or Boonfix, PAXgene tissue fixation is an alcohol based fixative and could therefore lead to further standardization of IHC [13] and histological procedures in general. Furthermore, prolonged fixation in PAXgene tissue fixative will not result in increased numbers of cross-links, an effect known to occur after prolonged formalin fixation, and one which often leads to false negative or doubtful IHC results and the resulting clinical impact [19]. Using not recommended alternative longer stabilization times showed that tissues fixed for 24 hours and than stored in stabilizer for 3 months, both morphology and antigenicity (CD31, KER-07, synaptophysin and chromogen A) remain intact. It was, however, necessary to mount the sections of long term stabilized fatty tissue on poly L-lysin coated slides to avoid loosing sections after antigen retrieval. It is, however, not common that tissue stored for that amount of time in stabilizer fluid (or presently formalin) is used for diagnostic purposes.

Although this work focuses on the morphological and immunohistochemical aspects of PAXgene tissue fixation, other members of the SPIDIA consortium have performed extensive investigations of the nature of proteins and nucleic acids in FFPE and PFPE tissues. Proteins derived from PFPE tissue show reactivity patterns analogous to proteins derived from frozen tissue, as opposed to proteins isolated from FFPE tissue which show decreased activity in proteomic testing [16]. Furthermore, when the properties of the new fixative with regard to stabilization of nucleic acids were investigated, preliminary data suggested that RNA is preserved far better in PAXgene-fixed as compared to formalin-fixed tissue. A detailed study will be reported in the near future by Viertler *et al.* (submitted for publication).

Although biomolecules appear to be preserved in a more native state, it is not yet known how long these molecules remain stable in PFPE tissue blocks. It is known that RNA deteriorates even after tissue is embedded in paraffin [20]. In FFPE tissue, the RNA is cross-linked and therefore of inferior quality to start with, but conversely, these cross-links provide stability. PAXgene fixation leaves RNA intact from the beginning, but since no cross-links are formed by PAXgene fixation, stability may be an issue [21]. Ongoing experiments on storage conditions and their effect on diverse tissue derivates will provide more insight into this matter.

Since the time when formalin replaced alcohol fixation over a century ago [22], analytical technologies were developed for use on formalin-fixed tissue. Nowadays, technology and medical care have reached a point at which a non-cross-linking fixative like PAXgene is once again preferable. Medical care demands standardized diagnoses and the use of a broader spectrum of diagnostic tools. Gene chips, which require high quality tissue RNA, have been developed and will one day be widely used for diagnostics [23]. Although genomic and proteomic techniques can be applied to biomolecules derived from FFPE tissue [3]–[8], the fixation time dependent variability of the number of cross-links in each tissue sample makes standardization, and therefore reproducibility, of downstream procedures virtually impossible. If standardization of formalin fixation were possible, it would have easily been established during the last century. The standardization and nucleic acid and protein friendly tissue fixation that the PAXgene fixative can offer may provide answers to these demands. Implementation of PAXgene fixation in routine pathology has one added advantage in that, as opposed to formalin fixation, PAXgene fixation reagents have no known carcinogenic effects (according to manufacturer's material safety data sheet). Proper air regulation as installed to avoid formalin toxicity, will keep alcohol toxicity levels to the required minimum. Therefore, introduction of this fixative could lead to the much desired formalin-free environment in pathology laboratories. Preliminary results obtained by fixation of whole pig organs show that, when tissue is pretreated as usual (e.g. cutting open to allow fixation) PAXgene is able to fix organs overnight. A beneficial side effect of the alcohol based fixation is that small lymph nodes embedded in fatty tissue are easily detectable by visual inspection. When specimens were stored in the stabilizer reagent for up to 3 months morphology remained optimally preserved. Furthermore, histology of tissues fixed in PAXgene for only 3 hours were comparable to that of tissues fixed in formalin or PAXgene for 24 hours indicating that eventual use of PAXgene in routine pathology could shorten the time from tissue acquisition to pathology result thus improving laboratory work flow. When tissue is placed in PAXgene tissue stabilizer, the fixation process stops while all tissue components are stable until processing. Potentially, the stabilization step could be performed in any processing machine so that the laboratory workflow does not need to be adapted to a two reagent system. PAXgene fixation of post mortem bone marrow samples showed that decalcification is still necessary, eventhough the fixative contains acetic acid. This decalcification step (10% formic acid 48 to 72 hours) did not influence morphology. Of course, before PAXgene can be fully implemented in routine pathology, more specific questions must be answered. While preparing this manuscript, tissue collection was ongoing to build a shadow archive of PFPE tissue. This archive will be subjected to all imaginable diagnostic testing and basic research techniques. A tissue atlas will be produced to serve as a reference manual for pathologists. When all tissue types are explored and described it will be more acceptable for pathologists to learn a new set of artefacts. Issues like biomolecule stability in this archive will be addressed, as well as the true versatility of the fixative in a clinical pathology setting. This stepwise introduction will build confidence in the fixative while exploring the molecular diagnostic and research possibilities. Only when trustworthy results are obtained in all fields of expertise can this fixative be implemented without obstacles.

In conclusion, PAXgene tissue fixation and stabilization reagents provide contemporary tissue related molecular medical research the best of both worlds. Although the technique is not without compromise, all currently assessed conventional histological techniques are fully applicable on PFPE tissue. Data produced within the SPIDIA project show that proteins and nucleic acids are preserved in a more native state in PFPE tissue compared to FFPE tissue [16]. We believe that after more extensive assessment in routine (molecular) pathology, the PAXgene fixation method can be used for primary diagnosis by routine H&E and IHC while enabling more accurate proteomic and molecular profiling for determination of prognosis and drug response.
